# A phase 1 trial of SGN-CD70A in patients with CD70-positive diffuse large B cell lymphoma and mantle cell lymphoma

**DOI:** 10.1007/s10637-018-0655-0

**Published:** 2018-08-22

**Authors:** Tycel Phillips, Paul M. Barr, Steven I. Park, Kathryn Kolibaba, Paolo F. Caimi, Saurabh Chhabra, Edwin C. Kingsley, Thomas Boyd, Robert Chen, Anne-Sophie Carret, Elaina M. Gartner, Hong Li, Cindy Yu, David C. Smith

**Affiliations:** 1 University of Michigan Comprehensive Cancer Center, 1500 E. Medical Center Dr. SPC 591, Ann Arbor, MI 48109 USA; 2 James P. Wilmot Cancer Center, University of Rochester Medical Center, 601 Elmwood Ave, Rochester, NY 14642 USA; 3 Levine Cancer Institute and Carolinas Healthcare System, 100 Medical Park Dr Ste 110, Concord, NC 28025 USA; 4 Northwest Cancer Specialists, P.C, 210 SE. 136th Ave, Vancouver, WA 98684 USA; 5 Case Western Reserve University, University Hospitals Cleveland Medical Center, 11100 Euclid Ave Ste 1, Cleveland, OH 44106 USA; 6 Medical College of Wisconsin, 9200 West Wisconsin Avenue, Milwaukee, WI 53226 USA; 7 Comprehensive Cancer Centers of Nevada, 3730 S. Eastern Ave, Las Vegas, NV 89169 USA; 8 Yakima Valley Memorial Hospital, North Star Lodge, 808 N 39 Ave, Yakima, WA 98902 USA; 9 City of Hope National Medical Center, 1500 East Duarte Rd, Duarte, CA 91010 USA; 10 Seattle Genetics, Inc., 21823 20th Dr SE, Bothell, WA 98021 USA

**Keywords:** Mantle-cell lymphoma, Diffuse, large B cell, lymphoma (DLBCL), Grade 3 follicular lymphoma, Antibody-drug conjugate, CD70 antigen, Pyrrolobenzodiazepine dimer (PBD)

## Abstract

*Purpose* This first-in-human study evaluated SGN-CD70A, an antibody-drug conjugate (ADC) directed against the integral plasma membrane protein CD70 and linked to a pyrrolobenzodiazepine (PBD) dimer, in patients with relapsed or refractory (R/R) CD70-positive non-Hodgkin lymphoma (NHL) including diffuse large B cell lymphoma (DLBCL), mantle cell lymphoma (MCL), and Grade 3b follicular lymphoma (FL3b). *Methods* SGN-CD70A was administered intravenously on Day 1 of 3-week cycles beginning at 8 mcg/kg with planned dose escalation to 200 mcg/kg. Due to observations of prolonged thrombocytopenia, the study was amended to dose every 6 weeks (q6wk). *Results* Twenty patients were enrolled and treated with SGN-CD70A. The maximum tolerated dose of SGN-CD70A was 30 mcg/kg q6wk. The most common adverse events (AEs) reported were thrombocytopenia (75%), nausea (55%), anemia (50%), and fatigue (50%). The onset for treatment-related thrombocytopenia typically occurred during Cycle 1. Most of the treatment-related events of thrombocytopenia were ≥ Grade 3. Antitumor activity in patients included 1 complete remission (CR) and 3 partial remissions (PRs), 2 of which were ongoing for at least 42.9 weeks. SGN-CD70A exposures were approximately dose proportional, with a mean terminal half-life of 3 to 5 days. *Conclusions* While modest single-agent activity was observed in heavily pretreated NHL patients, the applicability of SGN-CD70A is limited by the frequency and severity of thrombocytopenia, despite the long-term response with limited drug exposure.

**Electronic supplementary material:**

The online version of this article (10.1007/s10637-018-0655-0) contains supplementary material, which is available to authorized users.

## Introduction

Non-Hodgkin lymphoma (NHL) is a heterogeneous disease entity that has increased in prevalence over the last several decades. Based on the World Health Organization (WHO) classification of hematological and lymphoid tumors, NHL can be broadly classified as B or T/natural killer (NK) cell neoplasms [1].

There are multiple histological subtypes and gene-expression subgroups of NHL. The most common form of B cell lymphoma is diffuse large B cell lymphoma (DLBCL) [2]. DLBCL is an aggressive lymphoma characterized by large, abnormal B cell lymphocytes that no longer respond to growth-limiting signals in cell reproduction. While complete remission (CR) rates with frontline therapy in DLBCL have been reported to be as high as 75 to 80% [3], often these responses are not durable. For patients who do not achieve a CR in the frontline setting or who experience disease relapse, intensive chemotherapy followed by autologous stem cell transplant (SCT) is standard treatment. The 5-year survival rate after anthracycline-based chemotherapy ranges from 35 to 60% depending on gene-expression subgroups [4]. The introduction of rituximab into front-line therapies of NHL has greatly improved outcomes, but patients who have refractory or relapsed (R/R) disease still have very poor outcomes in spite of aggressive salvage therapy [5].

Although follicular lymphoma (FL) is generally chronic and incurable, Grade 3b FL (FL3b), is differentiated from other grades of FL by its aggressive nature and treatment paradigm, which is the same as for DLBCL. No standard treatments exist for patients with DLBCL or FL3b who are not eligible for or who have relapse/recurrence after SCT.

Mantle cell lymphoma (MCL), occurring in only 5 to 6% of all NHL cases, is another lymphoma that is considered incurable with current treatment modalities. The vast majority of patients relapse after frontline therapy and eventually become refractory to salvage treatment. For frontline treatment of MCL, an aggressive chemotherapy regimen is typically employed with an emphasis on regimens that include high dose cytarabine, such as: rituximab, hyperfractionated cyclophosphamide, vincristine, doxorubicin, and dexamethasone (R-HyperCVAD) [6]; dexamethasone, cytarabine, and cisplatin (RDHAP); or the Nordic Regimen consisting of intense immunochemotherapy (including rituximab) in patients who are considered eligible for autologous SCT [7]. Less intensive regimens such as bendamustine and rituximab are utilized in older or frail patients [8]. Newer agents, such as the Bruton’s tyrosine kinase (BTK) inhibitor ibrutinib, are associated with high response rates and good durability, but an unmet need remains for patients who have baseline resistance to or eventually fail this treatment [9].

CD70, the cellular ligand of the tumor necrosis factor receptor family member CD27, is expressed on a wide variety of malignancies including lymphoma. While its role is not completely understood, CD70 activation has been linked to proliferation and survival, raising the possibility that CD70, through its interaction with CD27, functions as an activation receptor on malignant B cells and an inhibitor of T cell response [10]. Given the high reported rate of expression in NHL (as high as 60% of NHL cases based on internal data), CD70 is an attractive therapeutic target for antibody-based therapy in this disease.

SGN-CD70A is a CD70-directed antibody-drug conjugate (ADC) consisting of 3 functional subunits composed of an anti-CD70 antibody, a protease-cleavable linker, and a DNA-crosslinking pyrrolobenzodiazepine (PBD) dimer drug. Upon SGN-CD70A binding to CD70, the complex internalizes and traffics to lysosomes where the delivered drug is released from the conjugate through proteolytic degradation of the dipeptide linker. The released PBD dimer crosslinks DNA, which initiates cellular events that lead to double strand breaks and eventual cellular apoptosis [11].

SGN-CD70A was evaluated in 2 separate cohorts in an initial dose finding study. We herein report the results of the high-risk NHL cohort. The objectives of this study were to determine the toxicity profile, maximum- tolerated dose (MTD), dose-limiting toxicities (DLTs), pharmacokinetic (PK) properties, preliminary anti-tumor efficacy, and a recommended phase 2 dose of SGN-CD70A for future studies.

## Patients and methods

### Patient eligibility

Eligible patients were ≥ 18 years of age and had a pathologically confirmed diagnosis of CD70-positive MCL or DLBCL including FL3b as determined by central pathology review (defined as expression in at least 50% of the sample) and radiographic evidence of disease. All patients were required to have been relapsed, refractory, or have progressive disease following at least 2 prior systemic therapies. Specifically, patients with DLBCL or FL3b were required to have previously received a multiagent chemoimmunotherapy regimen given with curative intent and must have received intensive salvage chemotherapy with SCT, unless deemed ineligible. Patients with MCL must have received a chemoimmunotherapy regimen. Patients could not have had prior allogeneic SCT within 100 days of enrollment. They could not have received prior anti-CD70-directed therapy unless CD70 expression was confirmed by central pathology review on a biopsy obtained after the treatment. Patients had an Eastern Cooperative Oncology Group (ECOG) performance status 0 or 1 with adequate baseline renal, hepatic, and bone marrow function. The original baseline platelet count criterion was amended from ≥75,000/μL to ≥100,000/μL to ensure adequate bone marrow function after observations of prolonged thrombocytopenia with SGN-CD70A treatment.

### Study design and treatment

This phase 1, open-label, dose-escalation study (NCT02216890) was designed to evaluate the safety and tolerability of SGN-CD70A and to establish the MTD in patients with CD70-positive R/R NHL and metastatic renal cell carcinoma (RCC). Results in patients with metastatic RCC will be reported separately. Nine centers in the United States enrolled patients with NHL between November 3, 2014 and November 13, 2015, under approval by an Institutional Review Board in accordance with the Declaration of Helsinki. All patients provided informed consent prior to administration of any study treatment.

The study initiated with a dosing schedule of SGN-CD70A administered intravenously (IV) on Day 1 of 3-week cycles, with planned dose levels for dose escalation of 8, 15, 30, 50, 80, 120, 160, and 200 mcg/kg. Due to prolonged thrombocytopenia, the study was amended to dose every 6 weeks (q6wk) to allow the bone marrow sufficient time to recover. This change in dosing schedule applied to patients already on study, as well as newly enrolling patients. Patients who achieved stable disease (SD) or better were eligible to continue receiving study treatment until disease progression or unacceptable toxicity.

The evaluation period to assess for DLTs was the first cycle of treatment. A DLT was defined as any clinically significant, non-hematologic adverse event (AE) ≥ Grade 3 according to the National Cancer Institute’s Common Terminology Criteria for Adverse Events (NCI-CTCAE), Version 4.03; non-hematologic laboratory abnormalities ≥ Grade 3 that did not resolve to ≤ Grade 1 or baseline within 1 day; ≥ Grade 4 neutropenia lasting more than 7 days; ≥ Grade 3 febrile neutropenia; Grade 4 thrombocytopenia; Grade 3 thrombocytopenia with bleeding, or any requirement for platelet transfusion; Grade 4 anemia unrelated to underlying disease; and delay of treatment by more than 7 days due to toxicity. The final MTD was determined by the Safety Monitoring Committee (SMC) and based on the MTD estimated by the modified continual reassessment method (mCRM) model along with all available safety data.

### Safety assessments

Safety assessments included the surveillance and recording of AEs and laboratory tests. Patients were required to undergo physical examination and assessment of AEs prior to each cycle of therapy. Laboratory evaluations were performed weekly throughout the first 6 cycles of treatment. AEs were summarized using the Medical Dictionary for Regulatory Activities (MedDRA), version 20.0. All edema-related events were consolidated using the Standardized MedDRA Query (SMQ) for angioedema. AEs and laboratory results were graded using the NCI CTCAE, version 4.03. A SMC monitored the safety of patients treated with SGN-CD70A on a regular basis throughout the study, including reviews of the data pertinent to dose-escalation decisions.

### Efficacy assessment

Imaging response was determined in Cycles 2, 4, and 7, then every third cycle for patients being treated every 3 weeks (q3wk). After the study was amended for q6wk dosing, response assessments were performed in Cycles 1, 2, and every other cycle thereafter. For patients who discontinued treatment without progression, evaluations continued every 12 weeks until progression or start of new anticancer therapy (with the exception of SCT). Investigators obtained spiral computed tomography (CT) scans of chest, neck, abdomen, and pelvis. Additionally, whole body positron emission tomography (PET) scans were required. Imaging response was determined based on the International Working Group Revised Response Criteria for Malignant Lymphoma [12].

The study was amended to require a bone marrow aspirate and biopsy at baseline to determine bone marrow involvement. If there was bone marrow involvement at baseline or if bone marrow involvement was unknown, bone marrow aspirate and biopsy were required to confirm CR. Patients who had an imaging response of SD or better at the same visit as investigator claim of clinical progression were counted as clinical progression for determination of best response. Patient’s best response, onset of response, and onset of disease control must have occurred prior to any subsequent therapies including SCT.

### Pharmacokinetic, pharmacodynamic, and immunogenicity assessments

In the q3wk dose cohorts, blood samples for SGN-CD70A PK analysis were collected predose; within 15 min after the end of infusion; and 2, 6, and 24 h, and 3, 7, 14, and 21 days from the start of infusion in Cycles 1, 2, and 4. In the q6wk dose cohorts, a blood sample for SGN-CD70A PK analysis was also collected 28 days from the start of infusion. In both dose cohorts, samples were collected only predose and within 15 min after the end of infusion in other cycles, and at the End of Treatment (EOT) visit. Blood samples for assessing the presence of anti-therapeutic antibody (ATA) were collected predose on Day 1 of the first 5 cycles, every fifth cycle thereafter, and at the EOT.

Sensitive, qualified assays were used to measure concentrations of ADC (SGN-CD70A), total antibody (TAb), and released-free drug, PBD, in plasma, and ATA in serum. The assays included enzyme-linked immunosorbent assays (ELISA) and liquid chromatography tandem mass spectrometry (LC MS/MS) assays. The limits of quantification for ADC, TAb, and PBD were 2.89 ng/mL, 2.93 ng/mL, and 10–20 pg/mL, respectively. PK parameters were estimated by non-compartmental analysis using Phoenix® WinNonlin® v6–3 (Certara, Princeton, NJ).

Blood samples were collected throughout the study to evaluate immune responses as appropriate.

### Statistical analysis

Descriptive statistics were used to summarize continuous variables. Frequencies and percentages were used to summarize categorical variables. Unless otherwise specified, confidence intervals (CIs) were calculated for a two-sided 95% level. Safety endpoints were summarized using the all-treated-patients set which included all patients treated with any amount of SGN-CD70A. All statistical analyses were performed using SAS® v9.4 (SAS Institute Inc. Cary, NC).

The number of patients with DLTs was summarized for all treated patients who either experienced a DLT or were followed for the full DLT evaluation period and did not receive growth factor or transfusion support. This study was conducted using a model-based mCRM that implemented Bayesian methodology to estimate the probabilities of DLT and response at each dose level. The dose-toxicity and dose-response relationship were modeled. The probability of DLT was estimated by the dose-toxicity model and the probability that the DLT rate is less than 30% was estimated for each dose level. In addition, the observed proportion of patients experiencing a DLT along with exact two-sided 95% CI using the Clopper-Pearson method [13] was reported where appropriate. The estimated MTD was the highest dose with an estimated DLT rate less than 30%. Additionally, a dose was defined as safe per the model if there was at least a 50% probability that the DLT rate was less than 30%.

All efficacy analyses are presented using all patients who receive any amount of SGN-CD70A. Objective response rate (ORR) and CR rate were also calculated using the efficacy-evaluable set that included all treated patients who had both a baseline and at least 1 evaluable post baseline disease assessment according to the Revised Response Criteria for Malignant Lymphoma [12] or per investigator claim of clinical progression. ORR was defined as the proportion of patients with CR or partial remission (PR). Duration of response (DOR) was defined as the time from start of the first documentation of objective tumor response (CR or PR) to the first documentation of disease progression or death due to any cause, whichever occurred first. DOR was only calculated for the subgroup of patients who achieved a CR or PR. Progression-free survival (PFS) was defined as the time from start of study treatment to the first documentation of disease progression or death due to any cause, whichever occurred first. All analyses were presented by assigned dose level and total except for time to event analysis. Since the number of patients in each dose level was small, PFS was analyzed combining all dose levels and using Kaplan Meier methodology. The two-sided 95% CI for median PFS was calculated using the complementary log-log transformation method [14].

The PK of SGN-CD70A ADC, TAb, and PBD (when measurable) were evaluated by noncompartmental analysis and summarized by descriptive statistics at each PK sampling time. ATA incidence rate was defined as the proportion of patients that developed ATA at any time during the study.

## Results

### Patients

Twenty patients were enrolled and treated with SGN-CD70A. Demographics and baseline disease characteristics are presented by dosing schedule in Table 1. The median age was 64.5 years (range, 28–81), 16 patients (80%) were male, and 19 patients (95%) were white. Most patients were diagnosed with DLBCL (not otherwise specified [NOS]) (9 patients [45%]) and MCL (5 patients [25%]) at study entry. The median number of prior systemic therapies was 3.5 (range, 2–8). All patients had an ECOG performance status of 0–1.

**Table 1:** Demographics and disease characteristics

	q3wk (*N* = 12)	q6wk (*N* = 8)	All treated (*N* = 20)
Median age in years (min, max)	63.5 (28, 81)	68.0 (54, 74)	64.5 (28, 81)
Male, n (%)	10 (83)	6 (75)	16 (80)
Race, n (%)			
White	11 (92)	8 (100)	19 (95)
American Indian or Alaska Native	1 (8)	0	1 (5)
ECOG performance status, n (%)			
0	5 (42)	3 (38)	8 (40)
1	7 (58)	5 (63)	12 (60)
NHL diagnosis subtype, n (%)			
T cell/histiocyte rich large B cell lymphoma	1 (8)	0	1 (5)
Mantle cell lymphoma	3 (25)	2 (25)	5 (25)
DLBCL (NOS)	7 (58)	2 (25)	9 (45)
Transformed DLBCL	0	2 (25)	2 (10)
Other	1 (8)	2 (25)	3 (15)
Number of prior systemic therapies per patient, n (%)			
Median	4.5	3.0	3.5
Min, Max	2, 8	2, 5	2, 8
Primary refractory disease, n (%)			
Yes	7 (58)	3 (38)	10 (50)
No	4 (33)	3 (38)	7 (35)
Missing	1 (8)	2 (25)	3 (15)
Current disease status, n (%)			
Refractory	6 (50)	3 (38)	9 (45)
Relapsed	5 (42)	3 (38)	8 (40)
Missing	1 (8)	2 (25)	3 (15)
History of bone marrow involvement, n (%)			
Yes	5 (42)	5 (63)	10 (50)
No	6 (50)	3 (38)	9 (45)
Missing	1 (8)	0	1 (5)
Current Bone Marrow Involvement, n (%)			
Yes	2 (17)	0	2 (10)
No	4 (33)	3 (38)	7 (35)
Unknown	6 (50)	5 (63)	11 (55)

*DLBCL*, diffuse large B-cell lymphoma; *ECOG*, Eastern Cooperative Oncology Group; *q3wk*, dose every 3 weeks; *q6wk*, dose every 6 weeks; *N*, number of treated patients; *NHL*, non-Hodgkin lymphoma

Twelve patients were treated q3wk and 8 patients were treated q6wk. Across both treatment schedules, half of the patients discontinued treatment due to progressive disease (10 patients [50%]); other reasons for discontinuation were AE (4 patients [20%]), investigator’s decision, and non-AE patient decision (3 patients [15%] each). AEs that led to treatment discontinuation were Grade 2 thrombocytopenia (2 patients [10%]), Grade 2 fluid retention (1 patient [5%]), and Grade 2 neutropenia (1 patient [5%]). One patient (5%) had a dose delay due to Grade 1 peripheral edema and 1 patient (5%) had a dose reduction due to Grade 3 increased lipase.

Both patients who discontinued due to thrombocytopenia reported more severe thrombocytopenia before and after the discontinuation event. The first patient had existing Grade 1 thrombocytopenia prior to the first dose of SGN-CD70A that worsened to Grade 4 approximately 2 weeks after the first dose. Although the event was Grade 2 at discontinuation, it worsened to Grade 3 and remained unresolved after discontinuation of treatment. The second patient experienced Grade 2 thrombocytopenia approximately 3 weeks after the first dose of SGN-CD70A. The event resolved to Grade 1, but worsened to Grade 3 after the second dose. The event remained unresolved at Grade 1 after discontinuation of treatment.

### Pharmacokinetics and immunogenicity

**Fig. 1:**
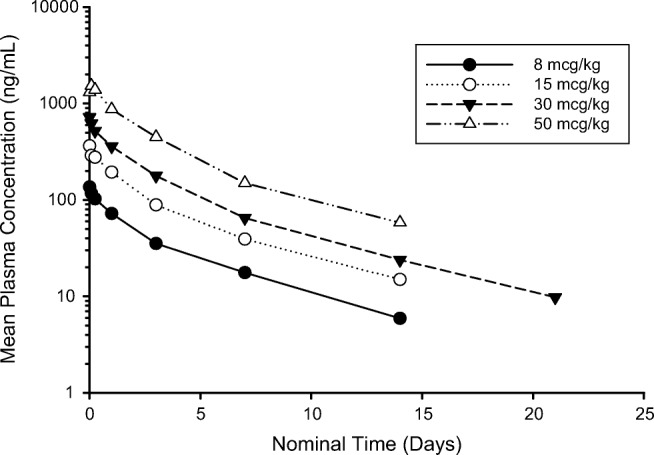
First-dose ADC mean concentration-time profile for patients treated with SGN-CD70A at 8, 15, 30 and 50 mcg/kg q3wk

PK parameters are summarized in Online Resource 1. Following IV administration of SGN-CD70A, plasma ADC concentrations appeared to decrease bi-exponentially with the mean terminal half-life (t_1/2_) between 3 and 5 days across the 8–50 mcg/kg dose levels for both q3wk (Fig. 1) and q6wk dosing schedules. After the first dose, the plasma ADC end-of-infusion concentration (C_eoi_) and exposure (area under the concentration-time curve from 0 to infinity [AUC_inf_]) were approximately dose-proportional. Consistent with the half-life, minimal accumulation was observed across cycles for SGN-CD70A ADC, except for approximately 30 to 40% higher exposure observed from Cycle 1 to Cycle 2 at 30 mcg/kg q3wk cohort.

Plasma TAb concentration-time profiles were similar to those of the ADC but the exposure of TAb was generally slightly higher. Plasma levels of the unconjugated cytotoxic agent, PBD, were below the lower limit of quantification (10–20 pg/mL) in all samples obtained from all patients at dose levels of 8–50 mcg/kg.

None of the ATA samples from all the treated patients (*N* = 20) were tested positive for anti-SGN-CD70A antibody at any visit during the study.

### Maximum-tolerated dose

Between the 2 dosing schedules, 19 patients were included in the DLT-evaluable set. A total of 3 patients (16%) had DLTs, 2 patients treated q3wk and 1 patient treated q6wk (Table 2). One DLT of Grade 3 elevated lipase was reported (Cycle 1 Day 8) in a patient who was treated q3wk at the 30 mcg/kg dose level. No associated pancreatitis symptoms or other significant lab abnormalities were observed. By Cycle 1 Day 15, the elevated lipase was reported to have recovered to Grade 1. The patient resumed treatment with a dose reduction and no recurrence was observed with continuing treatment at 15 mcg/kg. A second DLT of Grade 4 thrombocytopenia was reported (Cycle 1 Day 13) in a patient who was treated q3wk at the 50 mcg/kg dose level. In subsequent follow-up, Cycle 1 Day 35, the thrombocytopenia was reported to have improved to Grade 3. Another patient treated q6wk at the 50 mcg/kg dose level had a DLT of Grade 4 thrombocytopenia on Cycle 1 Day 17. In subsequent follow-up, Cycle 1 Day 29, the thrombocytopenia was reported to have improved to Grade 3. Neither of these patients who experienced thrombocytopenia received more than 1 dose of SGN-CD70A. No further information on resolution of these thrombocytopenia DLTs is available, since both patients were discontinued from the study. The MTD was determined to be 30 mcg/kg (DLT rate [standard deviation] = 0.164 [0.079]) with 93.9% probability of DLT rate being less than 30%.

**Table 2:** Summary of DLTs

	q3wk					q6wk			Total (N = 20)
	8 mcg/kg (N = 3)	15 mcg/kg (N = 3)	30 mcg/kg (*N* = 5)	50 mcg/kg (N = 1)	Subtotal (N = 12)	30 mcg/kg (*N* = 6)	50 mcg/kg (N = 2)	Subtotal (N = 8)	
Number of patients with DLTs, n(%)	0	0	1 (25)^a^	1 (100)	2 (18)^a^	0	1 (50)	1 (13)	3 (16)^a^
Adverse events considered as DLTs, n(%)									
Thrombocytopenia	0	0	0	1 (100)	1 (8)	0	1 (50)	1 (13)	2 (10)
Lipase increased	0	0	1 (20)	0	1 (8)	0	0	0	1 (5)

*DLT*, dose-limiting toxicity; *q3wk*, dose every 3 weeks; *q6wk*, dose every 6 weeks; *N*, number of treated patients

^a^Incidence was calculated based on DLT-evaluable patients (30 mcg/kg q3wk, *N* = 4; Subtotal q3wk, *N* = 11; Total, *N* = 19)

### Safety

The median duration of treatment for all patients treated with SGN-CD70A was 6 weeks (range, 3–27); the q3wk and q6wk dosing schedule durations were 6 weeks (range, 3–27) and 12.0 weeks (range, 6–18), respectively. The median number of doses for all patients was 2 (range, 1–8). Patients treated with 30 mcg/kg SGN-CD70A received a median of 3 doses (range, 1–4) and 2 doses (range, 1–3) in the q3wk and q6wk dosing schedules, respectively.

All 20 patients in the all-treated-patients set experienced at least 1 AE. The most common were thrombocytopenia (15 patients [75%]), nausea (11 patients [55%]), anemia (10 patients [50%]), and fatigue (10 patients [50%]) (Online Resource 2). Treatment-emergent AEs ≥ Grade 3 occurred in 18 total patients (90%); the most common AE ≥ Grade 3 was thrombocytopenia (13 patients [65%]; 7 patients treated q3wk and 6 patients treated q6wk). Other AEs ≥ Grade 3 that occurred in more than 1 patient (≥10%) were neutropenia (6 patients [30%]); anemia (5 patients [25%]); and congestive heart failure, *Clostridium difficile* infection, dyspnea, and decreased forced expiratory volume (2 patients [10%] each).

AEs considered to be related to SGN-CD70A were reported for a total of 16 patients (80%) (Table 3). The most common treatment-related AE for both the q3wk and q6wk schedules was thrombocytopenia, occurring in 7 of 12 patients and 6 of 8 patients, respectively, for a total of 13 patients (65%); most of these patients had an onset between Cycle 1 Day 15 to 22, regardless of dose or schedule. Most of the treatment-related events of thrombocytopenia were ≥ Grade 3 (12 patients [60%]). One of these patients experienced concurrent nose bleed and petechiae events (both Grade 1); there were no other bleeding events among these patients. Of the 22 thrombocytopenia events, 9 events (41%) resolved after a median of 2 weeks (range, 1.1 to 25.4) and 13 patients had unresolved thrombocytopenia at last follow-up. Median follow-up time for unresolved thrombocytopenia was 17.6 weeks (range, 0.1 to 71.0). Eight of the 12 patients who developed prolonged thrombocytopenia (Grade 3 or 4 for ≥7 days) had a history of bone marrow involvement. Of the 8 patients who did not develop thrombocytopenia (prolonged or otherwise), only 2 patients had a history of bone marrow involvement.

**Table 3:** Treatment-related AEs occurring in ≥20% patients in either treatment schedule

	q3wk (N = 12)		q6wk (N = 8)		Total (N = 20)	
Preferred term	Any grade	Grade ≥ 3	Any grade	Grade ≥ 3	Any grade	Grade ≥ 3
Any event	10 (83)	8 (67)	6 (75)	6 (75)	16 (80)	14 (70)
Thrombocytopenia	7 (58)	7 (58)	6 (75)	5 (63)	13 (65)	12 (60)
Anaemia	3 (25)	2 (17)	3 (38)	1 (13)	6 (30)	3 (15)
Neutropenia	3 (25)	2 (17)	3 (38)	3 (38)	6 (30)	5 (25)
Nausea	2 (17)	0	3 (38)	0	5 (25)	0
Fatigue	3 (25)	0	1 (13)	0	4 (20)	0
Oedema peripheral	2 (17)	0	2 (25)	1 (13)	4 (20)	1 (5)

*AE*, adverse event; *q3wk*, dose every 3 weeks; *q6wk*, dose every 6 weeks; *N*, number of treated patients

Consistent with the occurrence of thrombocytopenia, laboratory results in both dosing schedules reported ≥ Grade 3 low platelet counts: 6 of 12 (50%) patients treated q3wk and 5 of 8 (63%) patients treated q6wk. Two patients treated q3wk at the 50 mcg/kg and 30 mcg/kg dose level reported Grade 4 low platelet count values approximately 2 weeks after the first and second dose of SGN-CD70A, respectively.

Other treatment-related AEs across both treatment schedules included anemia (6 patients [30%]), neutropenia (6 patients [30%]), nausea (5 patients [25%]), fatigue (4 patients [20%]), and peripheral edema (4 patients [20%]). Of the 4 patients who reported treatment-related peripheral edema, 2 had Grade 1 and 1 patient each had Grade 2 and Grade 3. Two patients (10%) experienced treatment-related events of generalized edema; 1 of whom was among those with peripheral edema and also had Grade 1 hypoalbuminemia. A total of 9 patients (45%) experienced treatment-emergent edema events per the SMQ search strategy employed; 6 patients (50%) treated q3wk and 3 patients (38%) treated q6wk.

Of the 20 patients in the all-treated-patients set, 11 (55%) experienced at least 1 AE that was considered serious. The most frequently reported serious adverse events (SAEs) by preferred term were congestive heart failure, *Clostridium difficile* infection, and nausea, each reported by 2 patients (10%). All other events occurred in 1 patient each. Six patients (30%) experienced SAEs considered related to study treatment, 3 patients in each dosing schedule. SAEs reported for 1 patient each were adenocarcinoma of unknown primary, aplastic anemia, congestive heart failure, generalized edema, peripheral edema, pulmonary edema, and thrombocytopenia. The adenocarcinoma event was reported in a 74-year old patient diagnosed with MCL. Eighty-two days after the third and final dose of SGN-CD70A (30 mcg/kg; q6wk), the patient was hospitalized with bilateral pleural effusions; an analysis of pleural fluid was positive for adenocarcinoma. The patient was not treated for adenocarcinoma and did not develop any other evidence of adenocarcinoma; subsequent pleural biopsies and pleural effusion cytologies were found negative for adenocarcinoma. Due to the temporal association between the event of adenocarcinoma and administration of SGN-CD70A, a causal relationship could not be excluded.

Across both treatment schedules, 6 patients (30%) died while on study (4 patients treated q3wk; 2 patients treated q6wk); none of the deaths were within 30 days of the last dose of SGN-CD70A. Five patient deaths were disease-related. The sixth patient, with known cardiac risk factors including coronary artery disease, died of myocardial infarction 50 days after the first and only dose of SGN-CD70A (50 mcg/kg).

### Efficacy

**Fig. 2:**
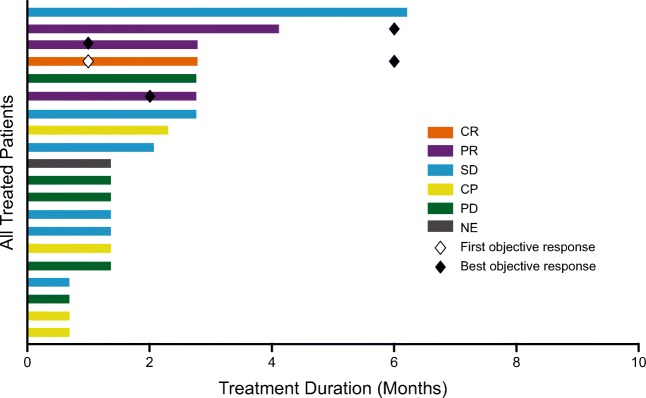
Duration on treatment by best response

The best response observed for all-treated-patients set is displayed in Table 4 by dosing schedule and the treatment duration is displayed in Fig. 2. The efficacy-evaluable set is the same as the all-treated-patients set. The ORR across both dosing schedules was 20% (4 of 20 patients [95% CI: 5.7, 43.7)]: 8% for patients treated q3wk (1 of 12 patients) and 38% for patients treated q6wk (3 of 8 patients). All of the patients who had a response were treated at the 30 mcg/kg dose level. One patient (5%) with transformed DLBCL, who received a total of 12.1 weeks of q6wk treatment, achieved a PR 5.1 weeks after starting treatment. Approximately 21 weeks after the last dose and without interim antineoplastic treatment, the patient achieved a CR and had an ongoing response at last follow-up (duration 36.1 + weeks).

**Table 4:** Summary of observed best response and PFS

	q3wk					q6wk			Total (N = 20)
	8 mcg/kg (N = 3)	15 mcg/kg (*N* = 3)	30 mcg/kg (N = 5)	50 mcg/kg (N = 1)	Subtotal (N = 12)	30 mcg/kg (N = 6)	50 mcg/kg (N = 2)	Subtotal (N = 8)	
Best Response^a^, n (%)									
Complete Remission (CR)	0	0	0	0	0	1 (17)	0	1 (13)	1 (5)
Partial Remission (PR)	0	0	1 (20)	0	1 (8)	2 (33)	0	2 (25)	3 (15)
Stable Disease (SD)	1 (33)	0	2 (40)	1 (100)	4 (33)	1 (17)	1 (50)	2 (25)	6 (30)
Progression	2 (67)	3 (100)	2 (40)	0	7 (58)	1 (17)	1 (50)	2 (25)	9 (45)
Progressive disease (PD)	1 (33)	3 (100)	0	0	4 (33)	1 (17)	0	1 (13)	5 (25)
Clinical Progression (CP)^b^	1 (33)	0	2 (40)	0	3 (25)	0	1 (50)	1 (13)	4 (20)
Not Evaluable (NE)	0	0	0	0	0	1 (17)	0	1 (13)	1 (5)
Objective response rate (CR + PR), n (%)	0	0	1 (20)	0	1 (8)	3 (50)	0	3 (38)	4 (20)
95% CI^c^ for objective response rate	(0.0, 70.8)	(0.0, 70.8)	(0.5, 71.6)	(0.0, 97.5)	(0.2, 38.5)	(11.8, 88.2)	(0.0, 84.2)	(8.5, 75.5)	(5.7, 43.7)
PFS									
Median duration in months (95% CI)									1.9 (1.1, −)

*CI*, confidence interval; *N*, number of treated patients; *PFS*, progression free survival

^a^CR, PR, SD and PD per Cheson 2007. CR, PR, SD, PD, CP and NE are mutually exclusive

^b^Patients with both PD and CP will be counted as PD. Patients who could not be assessed or were assessed as better than PD per Cheson, but had investigator claim of clinical progression at the same visit will be counted as CP

^c^Two-sided 95% exact CI, computed using the Clopper-Pearson method (1934)

A total of 3 patients (15%) achieved a best response of PR. The first patient had a PR at 5.6 weeks and received therapy for a total of 12.1 weeks (q3wk). The second patient treated q6wk had a PR at 27.1 weeks, approximately 15.3 weeks after the third and final dose administered. Both patients were known to have ongoing response at the last follow-up (durations of 42.9+ and 50.9+ weeks, respectively). The third patient had a PR at 8.1 weeks and received therapy q6wk for a total of 12 weeks. This patient had no further response assessment after the PR assessment was observed; therefore, no information is available regarding duration of this patient’s response.

**Fig. 3:**
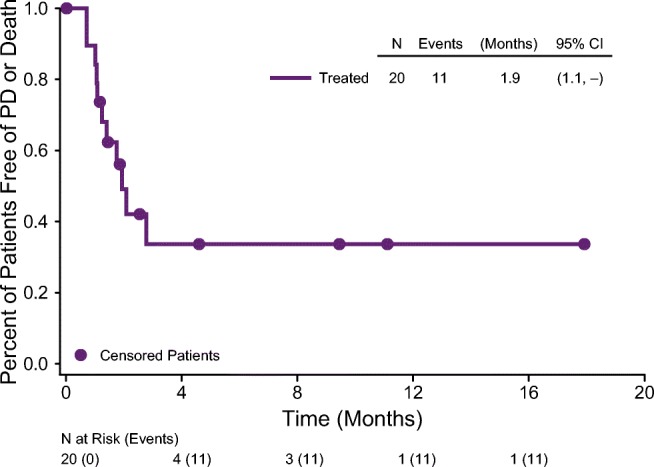
Kaplan-Meier plot of PFS

Six patients (30%) had a best response of SD: 4 of 12 treated q3wk and 2 of 8 treated q6wk. Nine patients (45%) had progression: 7 of 12 patients treated q3wk and 2 of 8 patients treated q6wk. By Kaplan Meier analysis, the estimated median PFS was 1.9 months (95% CI [1.1, −]) (Fig. 3).

## Discussion

Given the paucity of effective options for patients with R/R lymphomas, alternative treatments aside from traditional cytotoxic chemotherapy are needed. CD70 is an antigen that is expressed on a wide variety of cells, including NHL, with the potential to influence tumorigenesis making this an intriguing therapeutic target. In this open-label, single agent, dose-escalation phase 1 study, the ADC SGN-CD70A was evaluated in R/R DLBCL, including patients with disease that transformed from FL, and MCL. The treatment showed evidence of antitumor activity in this heavily-treated patient population with an ORR of 20%. Although the ORR is not remarkable on its own, 2 patients with PR were known to have ongoing responses at close to 1 year, despite no dose administration after approximately 12 weeks. While SGN-CD70A was able to be escalated to 50 mcg/kg, this was determined to be above MTD due to the occurrence of Grade 4 thrombocytopenia DLTs. As a result, improved antitumor activity at this and higher dose levels could not be further evaluated.

The most clinically meaningful toxicity observed was thrombocytopenia. After observation of prolonged recovery of the platelet count with dosing q3wk at both the 30 mcg/kg and 50 mcg/kg dose level, the protocol was amended to extend the dosing to q6wk. Additionally, a higher baseline platelet count was required for enrollment and baseline bone marrow exams were obtained to investigate if there was a correlation between bone marrow involvement and prolonged thrombocytopenia. Most patients with prolonged thrombocytopenia had a history of bone marrow involvement. In patients who received multiple infusions, the limiting AE of note was thrombocytopenia, indicating that SGN-CD70A could potentially be well tolerated over an extended treatment duration with mitigation of thrombocytopenia.

The mechanism of the observed thrombocytopenia is unknown. Several biomarkers were evaluated to determine a causative factor for the depth and duration of thrombocytopenia noted, including evaluation of Immunoglobulin G (IgG) antibody and thrombopoietin (TPO) levels. Neither of these analyses correlated with occurrence nor degree of thrombocytopenia noted. Furthermore, CD70 is not known to be expressed on megakaryocytes or its precursors. It is possible that the same mechanism that allowed for ongoing responses long after the last dose of SGN-CD70A also contributed to the ongoing thrombocytopenia.

Other AEs noted during this phase 1 study were fatigue and edema-related events. While fatigue is quite common for treatments evaluated in heavily-treated patient populations, the rate of edema-related events was an unexpected observation and its mechanism is unknown. No other dose-dependent trends were observed in the frequency or degree of the other AEs. We did not observe any significant infusion-related reactions during the DLT evaluation. Additionally, a high incidence of infectious complications was not noted during this study.

Taking into consideration the limited number of patients treated in the NHL cohort, the antitumor activity of the agent was reasonable with objective responses noted in 4 patients (20%). All responses were noted at the 30 mcg/kg dose with no responses noted at the 8, 15, or 50 mcg/kg dose, with the caveat that the 50 mcg/kg dosing level had limited exposure due to the incidence of thrombocytopenia. The responses noted at the 30 mcg/kg dose level were irrespective of the dosing schedule, with 1 PR noted on the q3wk dosing interval and 1 CR and 2 PRs on the q6wk dosing schedule. The majority of the responses were in patients with DLBCL (NOS), who comprised the majority of the study population. While all patients enrolled on study were required to have some degree of expression of CD70, there was no apparent correlation between response and level of expression in the patients. The median PFS was 1.9 months; however, 3 patients with follow-up after the response was observed had response durations of 36.1+, 42.9+, and 50.9+ weeks (responses ongoing at last follow-up).

**Fig. 4:**
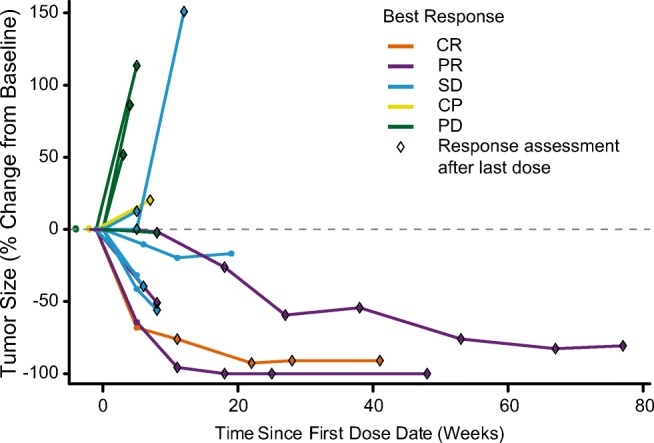
Percent change in sum of tumor diameters from baseline over time

It is notable that the majority of the patients enrolled on study, including those who responded, had limited exposure to SGN-CD70A, yet the patients who responded and were followed beyond EOT maintained the responses for a long duration (Fig. 4). This suggests that either the cytotoxic payload of SGN-CD70A, PBD, or CD70 target modulation may be associated with ongoing antitumor effects for a prolonged duration after exposure to treatment. In conjunction with prolonged thrombocytopenia, it is likely that the cytotoxic payload plays a larger role in this observation.

In conclusion, while CD70 would appear to be a promising target in the treatment of R/R NHL, the applicability of SGN-CD70A is limited by the frequency and severity of thrombocytopenia, despite the long-term of response with limited drug exposure. Given that we are currently unable to mitigate this AE, the rationale for further investigation of SGN-CD70A remains limited and is, therefore, not planned.

## Electronic supplementary material

Online Resource 1 (PDF 95 kb)

Online Resource 2 (PDF 93 kb)

## References

[1] Jaffe ES, Harris NL, Stein H, Campo E, Pileri SA, Swerdlow SH, Swerdlow SH, Campo E, Harris NL (2008). Chapter 8: introduction and overview of the classification of the lymphoid neoplasms. WHO classification of tumours of haematopoietic and lymphoid tissues.

[2] Flowers CR, Sinha R, Vose JM (2010). Improving outcomes for patients with diffuse large B-cell lymphoma. CA Cancer J Clin.

[3] Coiffier B, Lepage E, Briere J, Herbrecht R, Tilly H, Bouabdallah R, Morel P, Van Den Neste E, Salles G, Gaulard P, Reyes F, Lederlin P, Gisselbrecht C (2002). CHOP chemotherapy plus rituximab compared with CHOP alone in elderly patients with diffuse large-B-cell lymphoma. N Engl J Med.

[4] Rosenwald A, Staudt LM (2003). Gene expression profiling of diffuse large B-cell lymphoma. Leuk Lymphoma.

[5] Crump M, Neelapu SS, Farooq U, Van Den Neste E, Kuruvilla J, Westin J, Link BK, Hay A, Cerhan JR, Zhu L, Boussetta S, Feng L, Maurer MJ, Navale L, Wiezorek J, Go WY, Gisselbrecht C (2017). Outcomes in refractory diffuse large B-cell lymphoma: results from the international SCHOLAR-1 study. Blood.

[6] Romaguera JE, Fayad L, Rodriguez MA, Broglio KR, Hagemeister FB, Pro B, McLaughlin P, Younes A, Samaniego F, Goy A, Sarris AH, Dang NH, Wang M, Beasley V, Medeiros LJ, Katz RL, Gagneja H, Samuels BI, Smith TL, Cabanillas FF (2005). High rate of durable remissions after treatment of newly diagnosed aggressive mantle-cell lymphoma with rituximab plus hyper-CVAD alternating with rituximab plus high-dose methotrexate and cytarabine. J Clin Oncol.

[7] Geisler CH, Kolstad A, Laurell A, Andersen NS, Pedersen LB, Jerkeman M, Eriksson M, Nordstrom M, Kimby E, Boesen AM, Kuittinen O, Lauritzsen GF, Nilsson-Ehle H, Ralfkiaer E, Akerman M, Ehinger M, Sundstrom C, Langholm R, Delabie J, Karjalainen-Lindsberg ML, Brown P, Elonen E (2008). Long-term progression-free survival of mantle cell lymphoma after intensive front-line immunochemotherapy with in vivo-purged stem cell rescue: a nonrandomized phase 2 multicenter study by the Nordic lymphoma group. Blood.

[8] Rummel MJ, Al-Batran SE, Kim SZ, Welslau M, Hecker R, Kofahl-Krause D, Josten KM, Durk H, Rost A, Neise M, von Grunhagen U, Chow KU, Hansmann ML, Hoelzer D, Mitrou PS (2005). Bendamustine plus rituximab is effective and has a favorable toxicity profile in the treatment of mantle cell and low-grade non-Hodgkin's lymphoma. J Clin Oncol.

[9] Wang ML, Rule S, Martin P, Goy A, Auer R, Kahl BS, Jurczak W, Advani RH, Romaguera JE, Williams ME, Barrientos JC, Chmielowska E, Radford J, Stilgenbauer S, Dreyling M, Jedrzejczak WW, Johnson P, Spurgeon SE, Li L, Zhang L, Newberry K, Ou Z, Cheng N, Fang B, McGreivy J, Clow F, Buggy JJ, Chang BY, Beaupre DM, Kunkel LA, Blum KA (2013). Targeting BTK with ibrutinib in relapsed or refractory mantle-cell lymphoma. N Engl J Med.

[10] Claus C, Riether C, Schurch C, Matter MS, Hilmenyuk T, Ochsenbein AF (2012). CD27 signaling increases the frequency of regulatory T cells and promotes tumor growth. Cancer Res.

[11] Sandall S, Anderson M, Jonas M, Nesterova A, Miyamoto J, Stone IJ, Zeng W, Law C-L, Lewis TS (2014) SGN-CD70A, a novel and highly potent anti-CD70 ADC, induces double-strand DNA breaks and is active in models of MDR+ renal cell carcinoma (RCC) and non-Hodgkin lymphoma (NHL). Cancer res 74 (19 Suppl):abstract 2647

[12] Cheson BD, Pfistner B, Juweid ME, Gascoyne RD, Specht L, Horning SJ, Coiffier B, Fisher RI, Hagenbeek A, Zucca E, Rosen ST, Stroobants S, Lister TA, Hoppe RT, Dreyling M, Tobinai K, Vose JM, Connors JM, Federico M, Diehl V (2007). Revised response criteria for malignant lymphoma. J Clin Oncol.

[13] Clopper CJ, Pearson ES (1934). The use of confidence or fiducial limits illustrated in the case of the binomial. Biometrika.

[14] Collett D (1994) Interval-censored survival data. In: Collett D (ed) Modelling survival data in medical research, 1st edn. Chapman & Hall, London, pp 237–251

