# Supplementary material for: A phase 1 trial of SGN-CD70A in patients with CD70-positive diffuse large B cell lymphoma and mantle cell lymphoma
Source: Invest New Drugs. 2018 Aug 22;37(2):297–306. doi: 10.1007/s10637-018-0655-0 (PMC6440937; doi:10.1007/s10637-018-0655-0)
Supplement: Supplementary file 1 — (PDF 95 kb) [file 10637_2018_655_MOESM1_ESM.pdf]

Article Title: A phase 1 trial of SGN-CD70A in patients with CD70-positive Diffuse Large B Cell Lymphoma and Mantle Cell Lymphoma

Journal Name: Investigational New Drugs

Author Names: Tycel Phillips, Paul M. Barr, Steven I. Park, Kathryn Kolibaba, Paolo F. Caimi, Saurabh Chhabra, Edwin C. Kingsley, Thomas Boyd, Robert Chen, Anne-Sophie Carret, Elaina M. Gartner, Hong Li, Cindy Yu, David C. Smith

Correspondence: Tycel Phillips, MD
1500 E. Medical Center Dr. SPC 5911, Ann Arbor, MI 48109
Phone: (734) 647-8901
Fax: (734) 232-1328
Email: tycelp@med.umich.edu

Online Resource 1: First-dose pharmacokinetic parameters for SGN-CD70A ADC and TAb – q3wk and q6wk dosing schedule

	q3wk				q6wk	
	8 mcg/kg (N=3)	15 mcg/kg (N=3)	30 mcg/kg (N=5)	50 mcg/kg (N=1)	30 mcg/kg (N=6)	50 mcg/kg (N=2)
SGN-CD70A ADC						
AUC_{tau} (ng*day/mL)	406.18 (37) [n=2]	924.57 (54) [n=3]	1749.24 (30) [n=5]	4393.18 (-) [n=1]	1571.38 (26) [n=5]	1587.61 (10) [n=2]
AUC_{inf} (ng*day/mL)	414.02 (39) [n=2]	956.55 (56) [n=3]	1827.79 (33) [n=5]	4472.73 (-) [n=1]	1573.78 (26) [n=5]	1588.66 (10) [n=2]
C_{eoi} (ng/mL)	136.50 (6) [n=3]	360.07 (19) [n=3]	711.17 (17) [n=5]	1310.00 (-) [n=1]	737.81 (11) [n=6]	860.49 (32) [n=2]
$t_{1/2}$ (days)	3.46 (33) [n=2]	4.31 (28) [n=3]	5.15 (28) [n=5]	3.39 (-) [n=1]	3.94 (33) [n=5]	3.51 (10) [n=2]
V_{ss} (mL)	5594.22 (6) [n=2]	6749.76 (8) [n=3]	7669.83 (26) [n=5]	4577.79 (-) [n=1]	6472.24 (26) [n=5]	8777.73 (16) [n=2]
CL (mL/day)	1243.37 (44) [n=2]	1359.75 (31) [n=3]	1518.86 (40) [n=5]	1026.22 (-) [n=1]	1572.40 (38) [n=5]	2768.91 (29) [n=2]
SGN-CD70A TAb						
AUC_{tau} (ng*day/mL)	491.01 (-) [n=1]	1683.40 (3) [n=2]	2168.40 (36) [n=4]	6233.84 (-) [n=1]	2185.78 (37) [n=4]	2094.54 (-) [n=1]
C_{eoi} (ng/mL)	139.07 (12) [n=3]	376.88 (14) [n=3]	714.39 (17) [n=5]	861.00 (-) [n=1]	771.76 (17) [n=6]	782.19 (40) [n=2]

ADC, antibody-drug conjugate; AUC_{inf}, area under the concentration-time curve from 0 to infinity; AUC_{tau}, area under the concentration-time curve from 0 to 21 days for q3wk and from 0 to 42 days for q6wk; C_{eoi}, concentration at end of infusion; CL, clearance; q3wk, dose every 3 weeks; q6wk, dose every 6 weeks, $t_{1/2}$, terminal half-life, TAb, total antibody; V_{ss}, volume of distribution at steady state
Data are presented as geometric mean (% coefficient of variation).
