# Supplementary material for: A phase 1 trial of SGN-CD70A in patients with CD70-positive diffuse large B cell lymphoma and mantle cell lymphoma
Source: Invest New Drugs. 2018 Aug 22;37(2):297–306. doi: 10.1007/s10637-018-0655-0 (PMC6440937; doi:10.1007/s10637-018-0655-0)
Supplement: Supplementary file 2 — (PDF 93 kb) [file 10637_2018_655_MOESM2_ESM.pdf]

Article Title: A phase 1 trial of SGN-CD70A in patients with CD70-positive Diffuse Large B Cell Lymphoma and Mantle Cell Lymphoma

Journal Name: Investigational New Drugs

Author Names: Tycel Phillips, Paul M. Barr, Steven I. Park, Kathryn Kolibaba, Paolo F. Caimi, Saurabh Chhabra, Edwin C. Kingsley, Thomas Boyd, Robert Chen, Anne-Sophie Carret, Elaina M. Gartner, Hong Li, Cindy Yu, David C. Smith

Correspondence: Tycel Phillips, MD
1500 E. Medical Center Dr. SPC 5911, Ann Arbor, MI 48109
Phone: (734) 647-8901
Fax: (734) 232-1328
Email: tycelp@med.umich.edu

Online Resource 2: Treatment-emergent AEs occurring in ≥20% patients in either treatment schedule

	q3wk					q6wk			
Preferred Term	8 mcg/kg (N=3)	15 mcg/kg (N=3)	30 mcg/kg (N=5)	50 mcg/kg (N=1)	Subtotal (N=12)	30 mcg/kg (N=6)	50 mcg/kg (N=2)	Subtotal (N=8)	Total (N=20)
Any event, n (%)	3 (100)	3 (100)	5 (100)	1 (100)	12 (100)	6 (100)	2 (100)	8 (100)	20 (100)
Thrombocytopenia	1 (33)	2 (67)	4 (80)	1 (100)	8 (67)	5 (83)	2 (100)	7 (88)	15 (75)
Nausea	1 (33)	3 (100)	2 (40)	1 (100)	7 (58)	4 (67)	0	4 (50)	11 (55)
Anaemia	2 (67)	1 (33)	2 (40)	1 (100)	6 (50)	4 (67)	0	4 (50)	10 (50)
Fatigue	3 (100)	2 (67)	3 (60)	0	8 (67)	1 (17)	1 (50)	2 (25)	10 (50)
Neutropenia	1 (33)	0	2 (40)	1 (100)	4 (33)	2 (33)	1 (50)	3 (38)	7 (35)
Diarrhoea	1 (33)	1 (33)	1 (20)	1 (100)	4 (33)	1 (17)	1 (50)	2 (25)	6 (30)
Oedema peripheral	1 (33)	2 (67)	1 (20)	0	4 (33)	2 (33)	0	2 (25)	6 (30)
Abdominal pain	1 (33)	1 (33)	2 (40)	1 (100)	5 (42)	0	0	0	5 (25)
Constipation	1 (33)	2 (67)	0	1 (100)	4 (33)	1 (17)	0	1 (13)	5 (25)
Decreased appetite	1 (33)	0	2 (40)	0	3 (25)	2 (33)	0	2 (25)	5 (25)
Dyspnoea	0	2 (67)	0	1 (100)	3 (25)	1 (17)	1 (50)	2 (25)	5 (25)
Pyrexia	1 (33)	0	0	1 (100)	2 (17)	1 (17)	1 (50)	2 (25)	4 (20)

AE, adverse event; q3wk, dose every 3 weeks; q6wk, dose every 6 weeks
Treatment-emergent AEs are defined as newly occurring (not present at baseline) or worsening after first dose of investigational product.
